# Detecting drug-drug interactions between therapies for COVID-19 and concomitant medications through the FDA adverse event reporting system

**DOI:** 10.3389/fphar.2022.938552

**Published:** 2022-07-22

**Authors:** Eugene Jeong, Scott D. Nelson, Yu Su, Bradley Malin, Lang Li, You Chen

**Affiliations:** ^1^ Department of Biomedical Informatics, School of Medicine, Vanderbilt University Medical Center, Nashville, TN, United States; ^2^ Department of Biomedical Informatics, College of Medicine, the Ohio State University, Columbus, OH, United States; ^3^ Department of Biostatistics, School of Medicine, Vanderbilt University Medical Center, Nashville, TN, United States; ^4^ Department of Computer Science, School of Engineering, Vanderbilt University, Nashville, TN, United States; ^5^ Department of Computer Science and Engineering, College of Engineering, the Ohio State University, Columbus, OH, United States

**Keywords:** drug-drug interactions, COVID-19, FAERS, hypothesis generation, logistic regresion, additive interaction, multiplicative interaction, discovery-driven

## Abstract

**Background:** COVID-19 patients with underlying medical conditions are vulnerable to drug-drug interactions (DDI) due to the use of multiple medications. We conducted a discovery-driven data analysis to identify potential DDIs and associated adverse events (AEs) in COVID-19 patients from the FDA Adverse Event Reporting System (FAERS), a source of post-market drug safety.

**Materials and Methods:** We investigated 18,589 COVID-19 AEs reported in the FAERS database between 2020 and 2021. We applied multivariate logistic regression to account for potential confounding factors, including age, gender, and the number of unique drug exposures. The significance of the DDIs was determined using both additive and multiplicative measures of interaction. We compared our findings with the Liverpool database and conducted a Monte Carlo simulation to validate the identified DDIs.

**Results:** Out of 11,337 COVID-19 drug-Co-medication-AE combinations investigated, our methods identified 424 signals statistically significant, covering 176 drug-drug pairs, composed of 13 COVID-19 drugs and 60 co-medications. Out of the 176 drug-drug pairs, 20 were found to exist in the Liverpool database. The empirical *p*-value obtained based on 1,000 Monte Carlo simulations was less than 0.001. *Remdesivir* was discovered to interact with the largest number of concomitant drugs (41). *Hydroxychloroquine* was detected to be associated with most AEs (39). Furthermore, we identified 323 gender- and 254 age-specific DDI signals.

**Conclusion:** The results, particularly those not found in the Liverpool database, suggest a subsequent need for further pharmacoepidemiology and/or pharmacology studies.

## 1 Introduction

Coronavirus Disease 2019 (COVID-19) pandemic has continued to spread worldwide since late 2019. Over 2 years, after becoming a global pandemic, COVID-19 infection has been linked to over 267 million cases and over 5.2 million deaths worldwide as of 9 December 2021 (2021). Various antiviral medicines and other medications currently used in clinics with a well-established safety profile were repurposed for COVID-19 patients in order to prevent symptoms from deteriorating (Arshad et al., 2020; Wu et al., 2020). The United States Food and Drug Administration (FDA) approved one drug treatment (*remdesivir*) for COVID-19 and authorized others (e.g., *bamlanivimab* and *etesevimab*, *COVID-19 convalescent plasma*) for emergency use during this public health emergency and these drugs have shown promising efficacy against COVID-19 (Sheahan et al., 2017; Martinez, 2020; Mitja and Clotet, 2020). However, one of the primary concerns is drug safety, particularly with regard to drug-drug interactions (DDIs). Patients impacted by severe COVID-19 include not just the elderly, but also younger persons who are struggling with co-morbid conditions (Ji et al., 2020; Simonnet et al., 2020; Zhou et al., 2020). As many as 20–51% of affected COVID-19 patients have at least one comorbidity (Chen et al., 2020; Huang et al., 2020), and those patients are more likely to use polypharmacy, increasing the risk of adverse DDIs (Back and Marzolini, 2020). Additionally, a more complicated DDI is expected for COVID-19 patients because changes in the expression and activity of transporters and drug metabolizing enzymes (DMEs) during highly prevalent acute and chronic inflammatory conditions may alter the pharmacokinetics and pharmacodynamic properties of therapeutic drugs used in COVID-19 treatment (Mann, 2006). Although the prevalence of DDIs in COVID-19 patients has not been thoroughly evaluated, observational studies indicate that there are potential health risks associated with the use of repurposed drugs for COVID-19 treatments (Agency., 2020). For instance, several studies have recently been published which demonstrate that the combination of *hydroxychloroquine* and *azithromycin* increases the risk of QTc-time prolongation (Nguyen et al., 2020). Moreover, it was shown that patients on direct oral anticoagulants who were admitted with COVID-19 had significantly increased plasma concentrations due to interactions with antiviral agents used to treat COVID-19 (Testa et al., 2020).

All drugs, including those used to treat COVID-19, are subjected to rigorous safety and efficacy testing in clinical trials before their approval for use. However, while clinical trials are frequently used to demonstrate drug efficacy, they rarely detect all safety concerns prior to its use in the real world. This is because clinical trials involve a relatively small number of randomly selected participants for a short period. Certain adverse effects may manifest themselves only after these products have been used by a larger and more heterogeneous population, including those with concurrent diseases, over a long period of time. Nonetheless, premarketing clinical trial data on drug efficacy are generally more comprehensive and reliable, although complete safety profiles cannot be obtained (Amery, 1999). Moreover, the efficacy and safety concerns about the use of repurposed drugs in COVID-19 patients are unclear and should be assessed, as the safety profile of a drug for one indication cannot always be extrapolated to another indication, necessitating continual monitoring of adverse events. Thus, there is a need for pharmacovigilance studies, which provide a rapid, inexpensive, and real-time monitoring of emergent safety concerns via the analysis of spontaneous reporting systems (SRS).

A primary goal of pharmacovigilance is to detect new adverse events (AEs) or changes in the incidence of AEs previously associated with the drugs in question, referred to as signal detection. Prior to the advent of powerful computer technology, signal detection relied solely on a qualitative approach or case-by-case analysis, implying that each incoming case report of a suspected AE submitted to a spontaneous reporting system was evaluated by an expert assessor (Wysowski and Fourcroy, 1996; Bennett et al., 2002). Despite the fact that the qualitative approach has been shown to be effective, as data availability and the complexity of drug–AE associations (i.e., DDIs) has increased, quantitative approaches to analyzing disproportionately abundant adverse events have become valuable in addition to qualitative signal detection. In contrast to hypothesis testing, which uses estimates to indicate the strength of interaction, quantitative approaches in SRS are primarily used for hypothesis generation, i.e., to find potential DDI signals supporting the need for future research to confirm associations between drugs. Several studies have used quantitative approaches to detect suspected AEs and to generate hypotheses about new AEs in real-world settings, and the SRS database has proven to be a useful source of evidence in the safety evaluation process (Ibrahim et al., 2016; Chasioti et al., 2019; Yao et al., 2020). Especially, the US Food and Drug Administration’s Adverse Event Reporting System (FAERS) database, which was created to support the FDA’s post-marketing safety surveillance program for drugs, has the advantage of being updated quarterly and including millions of reports, which is critical when investigating rare events.

In this study, we examined the potential DDIs in COVID-19 patients using a quantitative approach based on the FAERS database to evaluate the real consequences of potential DDIs. Furthermore, we examined age and gender disparities in DDI-related AEs.

## 2 Materials and methods

### 2.1 FAERS database

FAERS is a database that contains approximately 20 million reports of spontaneous AEs submitted by pharmaceutical companies, clinicians, pharmacists, and patients. It contains the following types of data: demographic (e.g., age and gender) and administrative information; drug names; AEs; patient outcomes (e.g., death, life-threatening, and disability); report sources; therapy dates; and indications for use (e.g., COVID-19). Were used. Each report refers to a single patient and may include one or more indications, drugs, and AEs. The AEs and indications in the FAERS database are coded using the Medical Dictionary for Regulatory Activities (MedDRA) Preferred Terms (PT), each of which is a single medical concept for a symptom, sign, disease diagnosis, therapeutic indication, investigation, etc. Our study used 21 months (January 2020—September 2021) of demographic (DEMOyyQq.TXT), drug (DRUGyyQq.TXT), indication for use (INDIyyQq.TXT), and outcome (OUTCyyQq.TXT) files in the FAERS database.

The FAERS database requires significant curation before it can be used effectively, and the procedures used to clean and normalize the data can have a considerable effect on the analytic outcomes. For instance, drug names in the FAERS database have not been curated, resulting in a number of ambiguous drug names that may be misclassified or misinterpreted. The Adverse Event Open Learning through universal Standardization (AEOLUS) (Banda et al., 2016) has been widely used and has been demonstrated to be a valuable solution for cleaning and normalizing the FAERS database. In this study, the AEOLUS was used to eliminate duplicate case records and apply standardized vocabularies by mapping drug names to RxNorm ingredients. Among 10, 758, 911 drug names, we achieved 96% coverage (10, 119, 657 drug names were mapped to RxNorm ingredients). We found that the remaining 4% (639,254 drug names) were not mapped because some of them lacked RxNorm ingredient information, while others had non-specific drug names, so we excluded those drug names. Our drug name mappings account for more than 99% of the 21-months reports found in the FAERS database.

### 2.2 COVID-19 and concomitant medications

To extract reports involving COVID-19 from the FAERS database, we used the following narrow Standardized MedDRA Query (SMQ) in the indication field: *SARS-CoV-2 test positive, COVID-19, exposure to SARS-CoV-2, COVID-19 pneumonia, symptomatic COVID-19, occupational exposure to SARS-CoV-2, COVID-19 treatment, Coronavirus test positive, coronavirus infection, COVID-19 immunization, COVID-19 prophylaxis, exposure to SARS-CoV-2, SARS-CoV-2 antibody* test positive, SARS-CoV-2 carrier, SARS-CoV-2 sepsis, SARS-CoV-2 - test false negative, and SARS-CoV-2 viraemia.

To avoid duplication of clinically similar AEs in COVID-19 reports, we grouped MedDRA PTs into their primary MedDRA High-Level Terms (HLTs). We note that MedDRA PTs are grouped into HLTs, which are grouped in High-Level Group Terms (HLGTs), which are included in indicating specific System Organ Classes (SOCs). We excluded MedDRA HLTs that were included in the following six SOCs: Injury, poisoning, and procedural complications; Social circumstances; Product issues; Surgical and medical procedures; Investigations; General disorders, and administration site conditions. These SOCs contain nonspecific disorders, laboratory test results, social issues, and therapeutic procedures, which are not the focus of our study.

To investigate COVID-19 DDIs, we partitioned the drugs into two groups: COVID-19 drugs contained only drugs used to treat the COVID-19; Co-medications included COVID-19 drugs, as well as drugs used to treat other disorders. We selected COVID-19 drugs using the criteria from Hodge et al. (Hodge et al., 2020), which selected experimental COVID-19 drugs based on a search of ClinicalTrials.gov. Their evaluation panel comprised of pharmacists, pharmacologists, and infectious diseases specialists discussed potential inclusion for all candidates identified. The list of experimental COVID-19 therapies is periodically updated and as of 7 February 2022, 34 drugs had been advanced for DDI analysis: *Anakinra, aspirin, azithromycin, bamlanivimb (alone), bamlanivimab/etesevimab, baricitinib, budesonide, canakinumab, casirivimab/imdevimab, CD24Fc immunomodulator, chloroquine, colchicine, convalescent plasma, COVID-19 vaccine, dalteparin, dexamethasone, enoxaparin, etesevimab (alone), favipiravir, fluvoxamine, hydrocortisone, hydroxychloroquine, infliximab, interferon beta, ivermectin, methylprednisolone, molnupiravir, niclosamide, nirmatrelvir/ritonavir, nitazoxanide, prednisolone, prednisone, remdesivir, ribavirin, ruxolitinib, sarilumab, sotrovimab, tixagevimab/cilgavimab, and tocilizumab.* The number of co-medications was 1,420.

### 2.3 DDI analysis

Analyses were performed and figures were generated using the open-source scripting language *Python* (version 3.8.10) and R (version 4.2.0).

The presence of statistical interaction indicates that the concurrent use of two drugs confers an increased risk of AE beyond that caused by each of the two drugs alone. Several statistical algorithms have been proposed to detect DDI signals from spontaneous reporting systems (Noguchi et al., 2019; Jeong et al., 2022), and previous studies investigated the detection tendency of models (Noguchi et al., 2020). However, there is no *de facto* standard for DDI signal detection models. Among several methods for detecting DDI, logistic regression has the benefit of adjusting for covariates. Confounders are hidden factors that may be responsible for falsely flagging critical DDI signals. For instance, males and females respond differently to drug treatment (Farkouh et al., 2020), and elderly patients are more susceptible to severe drug interactions than younger patients (Egger et al., 2007). We account for such confounding variables using logistic regression to detect more reliable DDIs.

The additive and multiplicative are the two most frequently used models to estimate DDIs. We applied an approach by incorporating logistical regression into additive and multiplicative models (VanderWeele and Knol, 2014). In the approach, we calculated covariate-adjusted additive and multiplicative interactions from the logistic regression. On an additive scale, interaction indicates that the combined effect of two drugs is greater than the sum of their individual effects, whereas, on a multiplicative scale, interaction indicates that the combined effect is greater than the product of their individual effects. Since reporting interaction measures on both additive and multiplicative scales is generally recommended because both can be informative (VanderWeele and Knol, 2014), we assessed interactions on both the additive and multiplicative scales. Our DDI detection framework is made up of three components: 1) logistical regression, which is to adjust for covariates; 2) relative excess risk due to interaction (RERI), which is to assess the additive interaction of two drugs; and 3) multiplicative interaction. We determined DDIs signals as there was evidence of an interaction on at least one scale. Furthermore, we assessed whether there existed differences in detected DDIs signals between patient groups divided by age and gender.

#### 2.3.1 Logistic regression

We used a penalized likelihood-based method called Firth logistic regression (Heinze and Schemper, 2002) with the occurrence of AEs as the dependent variable and the use of COVID-19 drugs and co-medications as the independent variables as follows:

$$\begin{array}{c} Logit\left[P\left(AE=1\right)\right]=\beta_{0}+\beta_{1}COVID-19\;Drug+\beta_{2}Co-medication\\ +\beta_{3}COVID-19\;Drug*Co-medication+\;\beta_{4}covariates\; \end{array}$$ (1)

When the number of events per variable (EPV) is small, the logistic regression is imprecise and biased (Jewell, 1984; Nemes et al., 2009). As such, we used the Firth logistical regression with a sample size criterion of 5 EPV. Firth’s penalization has gained increasing popularity to reduce the small-sample bias of maximum likelihood estimations (MLEs) by adding a penalty term that removes the first-order term in the asymptotic bias expansion of MLEs. Our model was adjusted with confounding factors, including age, gender, and the number of unique drug ingredient exposures.

#### 2.3.2 Additive interaction

**FIGURE 1:**
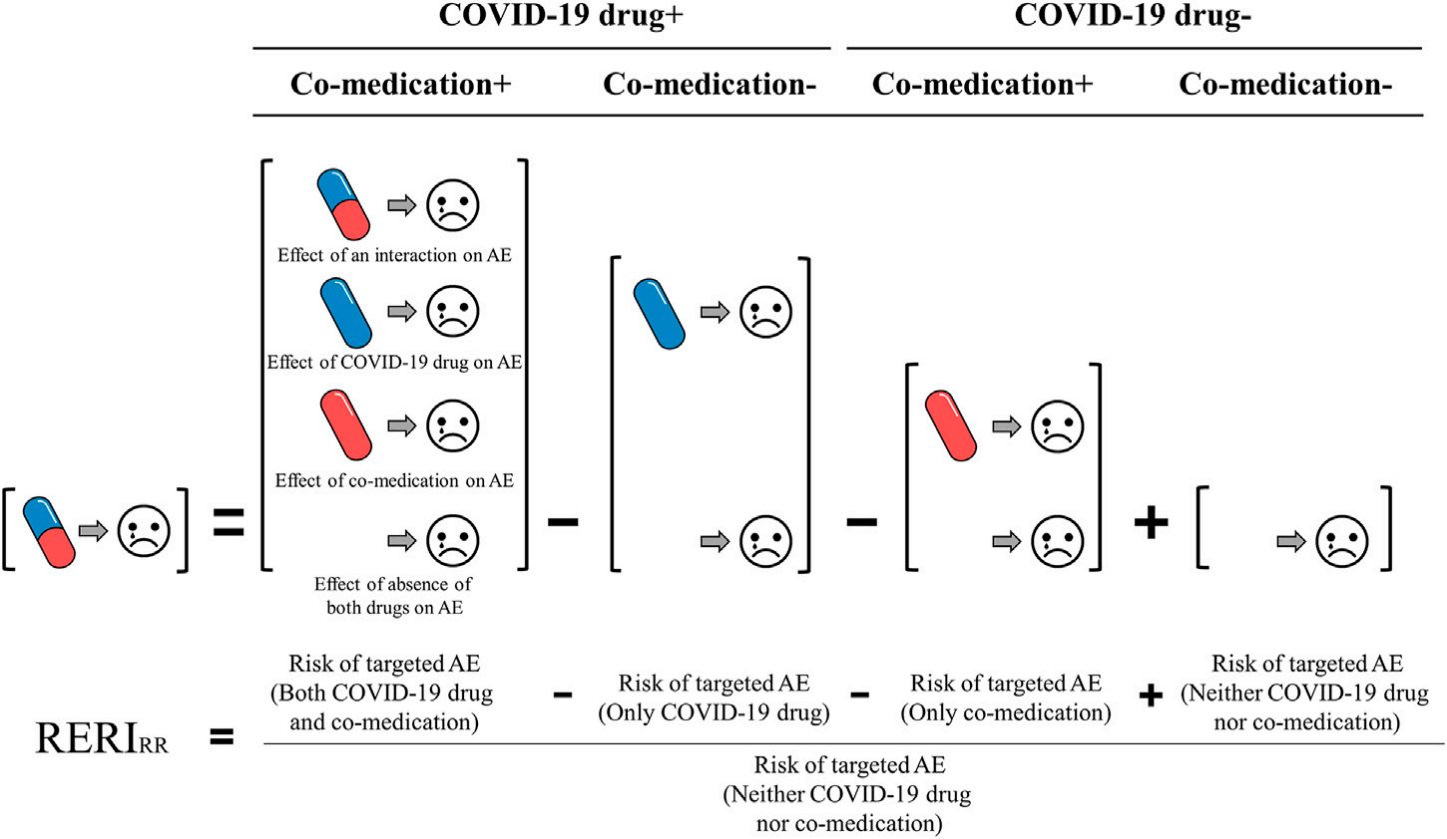
A measure of additive interaction between a COVID-19 drug and a co-medication on a targeted AE.

The RERI is defined as the increased risk due to the interaction given as the difference between the effect of the combination of a COVID-19 drug and a co-medication and the effect based on the sum of individual effects of a COVID-19 drug and a co-medication (Schottenfeld, 2002) (See Figure 1). The RERI has become a widely used metric for determining whether or not effects are additive (Hallan et al., 2006; Yang et al., 2009), which is defined as:

$$RERI_{RR}=RR_{11}-RR_{10}-RR_{01}+1$$ (2)

where $RR_{11}$ is the relative risk of developing a targeted AE if both a COVID-19 drug and a co-medication are present, $RR_{10}$ is the relative risk of developing a targeted AE if a COVID-19 drug is present but a co-medication is absent, and $RR_{01}$ is the relative risk of developing a targeted AE if a co-medication is present but a COVID-19 drug is absent.

However, since FAERS is a cross-sectional database containing self-reported AEs and potentially associated drugs, FAERS does not provide for a denominator (that is, how many people are actually taking the drug), such that the incidence is unknown. This limitation necessitates the use of a case-control study design. Since the relative risk is the measure of association in a cohort study and cannot be estimated directly in a case-control study design, we cannot directly estimate the $RERI_{RR}$ in our study design. However, for rare outcomes (typically, occurring in less than 10% of the study population), the odds ratios provide a reasonable approximation of the risk ratios (Greenland and Thomas, 1982; Shapiro, 1982), indicating that we could replace each of the risk ratios in $RERI_{RR}$ with odds ratios to obtain approximations to each of these measures of additive interaction, as follows:

$$RERI_{OR}=OR_{11}-OR_{10}-OR_{01}+1\;\approx RR_{11}-RR_{10}-RR_{01}+1=RERI_{RR}$$ (3)

Since confounders need to be identified and adjusted from the observed marginal associations (Greenland and Morgenstern, 2001; Brookhart et al., 2010; Harpaz et al., 2012), we obtained estimates of $RERI_{OR}$ based on the logistic regression model. $RERI_{OR}$ can be estimated using the parameters in Equation 1, described as follows:

$$RERI_{OR}=OR_{11}-OR_{10}-OR_{01}+1\;=\;e^{\left(\beta_{1}+\beta_{2}+\beta_{3}\right)}-\;e^{\beta_{1}}-e^{\beta_{2}}+1\;$$ (4)

The delta method was used to calculate the 95% confidence intervals of $RERI_{OR}$ (Assmann et al., 1996). $RERI_{OR}=0$ indicates that there is no interaction or that it is exactly additivity; $RERI_{OR}>0$ indicates that there is positive interaction or that there is more than additivity; $RERI_{OR}<0$ indicates that there is negative interaction or that there is less than additivity (VanderWeele and Robins, 2007; Rothman et al., 2008; VanderWeele and Knol, 2011). Thus, DDIs were considered significant in our study when both the estimate and 95% confidence interval lower bound for $RERI_{OR\;}$ are greater than 0.

### 2.3.3 Multiplicative interaction

The presence of interaction on the multiplicative scale for odds ratio was tested as follows:

$$\frac{OR_{11}}{OR_{10}OR_{01}}=e^{\beta_{3}}$$ (5)

Multiplicative interaction was evaluated using Equation 1 interaction effect $e^{\beta_{3}}$ , which is equivalent to $\frac{OR_{11}}{OR_{10}OR_{01}}$ , with a value greater than 1 and a *p*-value less than 0.05 indicating a DDI.

### 2.3.4 Age and gender disparities in AEs

Age was partitioned into two groups: younger than 65 and older or equal to 65, while gender was stratified into females and males. Odds ratios were calculated and *p*-values less than 0.05 were considered age- or gender-specific DDIs.

To interpret the DDI results, RxNorm ingredients were mapped to 1st level Anatomical Therapeutic Chemical (ATC) classes using the RxNorm API for drug classification purposes. The ATC classification, which was developed by the World Health Organization’s (WHO) Collaborating Centre for Drug Statistics Methodology, categorizes drugs into five levels, the first four of which correspond to anatomical, therapeutic, pharmacological, and chemical groups, and the fifth of which includes the actual drugs. We included the ATC 1st level, which contains 14 major anatomical or pharmacological groups, in our analysis of the findings. If a RxNorm ingredient had multiple ATC 1st level codes, all ATCs were counted separately. The COVID-19 drugs were assigned to the COVID-19 category rather than ATC 1st level. Also, MedDRA SOC was included to illustrate which adverse event categories were associated with interactions.

### 2.4 COVID-19 DDIs evaluation

The Liverpool database was used to assess which COVID-19 DDIs identified in our study were already known or novel. The Liverpool database is an open database based on Covid19-druginteraction.org developed by the University of Liverpool (Hodge et al., 2020) that assesses the risk of DDI associated with experimental COVID-19 therapies and is freely available to healthcare workers, patients and researchers. It is updated regularly as new treatment regimens for COVID-19 emerge, and the last update was performed on 13 June 2022. The Liverpool database contains information on the safety of combining COVID-19 drugs with concomitant drugs. The Liverpool database assessed DDIs using clinical pharmacology data extracted from approved product labels, published submissions to regulatory authorities, published case reports or studies, and, when none of the above were available, personal communication with the manufacturer. The risk of drug interaction was evaluated according to known pharmacokinetics (i.e., drugs involved against known Absorption, Distribution, Metabolism, and Excretion (ADME) pathway), overlapping toxicities, and QT risk. DDIs were graded into four levels: *no interaction, potential weak interaction*, *potential interaction, and do not co-administer.* DDIs were defined as interactions that did not fall into the “no interaction” category. It is updated regularly as new treatment regimens for COVID-19 emerge, and the last update was performed on 13 June 2022.

### 2.5 Monte Carlo simulation

We calculated an empirical *p*-value using 1,000 Monte Carlo simulations. We generated random drug-drug pairs with the same number of our significant drug-drug pairs in each simulation by shuffling between COVID-19 drugs and co-medications for each simulation. We calculated the empirical *p*-value as $p=\frac{\left(r+1\right)}{\left(n+1\right)}$ , where *r* is the number of times that random drug-drug pairs contained more or an equal number of confirmed DDIs (as determined by the Liverpool database) than our drug-drug pairs*,* and n is the number of replicate samples that have been simulated, which is 1,000 (North et al., 2002).

## 3 Results

### 3.1 Clinical and demographic characteristics of the patients

Table 1 summarizes the characteristics of the study population. There were 18,589 adverse reports, comprising 937 MedDRA HLTs and 1,420 RxNorm ingredients. After applying an EPV of 5 as a minimal guideline criterion, the COVID-19 cohort contained 10,195 COVID-19 drugs-co-medications-AE combinations. The female: male ratio was 47.3:52.7 and 9,991 reports were made by patients aged younger than 65 (55.6%). *Hydroxychloroquine* was the most frequently used drug (3,414 [18.4%]), followed by *remdesivir* (3,270 [17.6%]) and *azithromycin* (2,872 [15.5%]).

**TABLE 1:** Summary characteristics of the study population in the FAERS database from January 2020 to September 2021.

	Adverse event reports involving at least one COVID-19 drug (*n* = 18,589)		
	Female *n* (%)	Male *n* (%)	Total *n* (%)
Age			
<65	4,971 (26.74)	5,020 (27.01)	9,991 (53.75)
≥65	3,819 (20.54)	4,779 (25.71)	8,598 (46.25)
Number of drug exposures			
≤2	4,059 (21.84)	4,152 (22.34)	8,211 (44.17)
3–4	1,575 (8.47)	1,923 (10.34)	3,498 (18.82)
≥5	3,156 (16.98)	3,724 (20.03)	6,880 (37.01)
COVID-19 treatments			
Hydroxychloroquine	1,252 (6.74)	2,162 (11.63)	3,414 (18.37)
Remdesivir	1,293 (6.96)	1,977 (10.64)	3,270 (17.59)
Bamlanivimab	1,134 (6.1)	1,607 (8.6)	2,741 (14.7)
Azithromycin	971 (5.22)	1,901 (10.23)	2,872 (15.45)
Enoxaparin	786 (4.23)	1,226 (6.6)	2,012 (10.82)
Dexamethasone	779 (4.19)	1,230 (6.62)	2,009 (10.81)
Tocilizumab	499 (2.68)	1,211 (6.51)	1,710 (9.2)
Casirivimab/Imdevimab	721 (3.88)	641 (3.45)	1,362 (7.33)
Aspirin	509 (2.74)	762 (4.1)	1,271 (6.84)
Methylprednisolone	404 (2.17)	771 (4.15)	1,175 (6.32)
Prednisone	322 (1.73)	462 (2.49)	784 (4.22)
Bamlanivimab/Etesevimab	225 (1.21)	169 (0.91)	394 (2.12)
Baricitinib	117 (0.63)	161 (0.87)	278 (1.5)
Prednisolone	133 (0.72)	140 (0.75)	273 (1.47)
Hydrocortisone	88 (0.47)	164 (0.88)	252 (1.36)
Convalescent Plasma	76 (0.41)	131 (0.7)	207 (1.11)
Favipiravir	68 (0.37)	129 (0.69)	197 (1.06)
Budesonide	99 (0.53)	68 (0.37)	167 (0.9)
Anakinra	54 (0.29)	102 (0.55)	156 (0.84)
Chloroquine	42 (0.23)	108 (0.58)	150 (0.81)
Ivermectin	35 (0.19)	51 (0.27)	86 (0.46)
Sarilumab	31 (0.17)	51 (0.27)	82 (0.44)
Colchicine	28 (0.15)	48 (0.26)	76 (0.41)
Ribavirin	26 (0.14)	37 (0.2)	63 (0.34)
Interferon-Beta	14 (0.08)	42 (0.23)	56 (0.3)
Infliximab	28 (0.15)	25 (0.13)	53 (0.29)
Dalteparin	7 (0.04)	38 (0.2)	45 (0.24)
Canakinumab	9 (0.05)	21 (0.11)	30 (0.16)
Ruxolitinib	13 (0.07)	13 (0.07)	26 (0.14)
Fluvoxamine	9 (0.05)	8 (0.04)	17 (0.09)
Casirivimab	2 (0.01)	6 (0.03)	8 (0.04)
Sotrovimab	2 (0.01)	6 (0.03)	8 (0.04)
Etesevimab	2 (0.01)	0 (0)	2 (0.01)
Nitazoxanide	0 (0)	2 (0.01)	2 (0.01)
CD24Fc	0 (0)	0 (0)	0 (0)
leronlimab	0 (0)	0 (0)	0 (0)
Niclosamide	0 (0)	0 (0)	0 (0)

### 3.2 Potential DDIs

**FIGURE 2:**
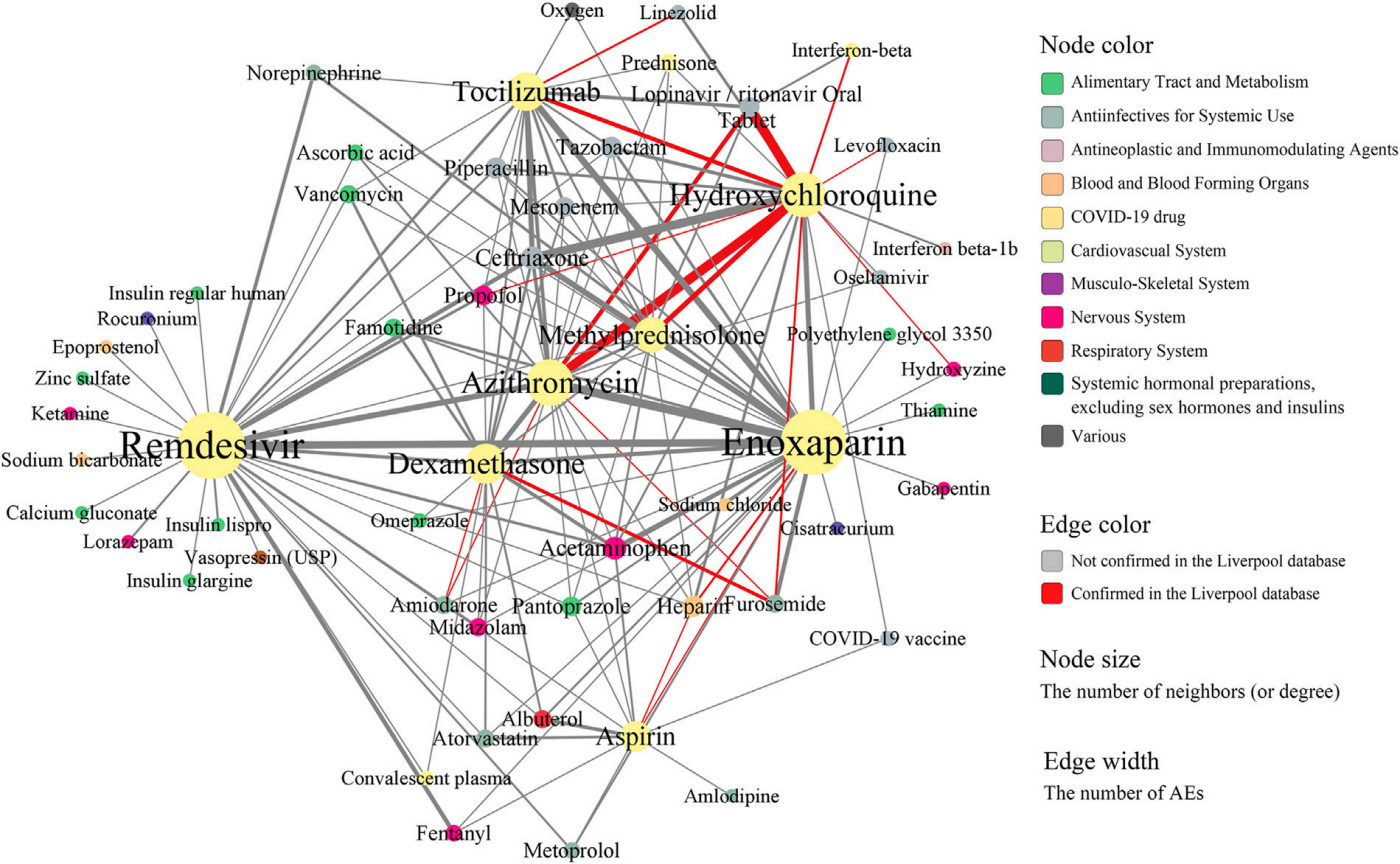
Drug-drug interaction network. The size of a node is proportional to the number of neighboring drugs, while the color corresponds to the ATC 1st level. The width of an edge is proportional to the number of unique AEs, while its color indicates whether or not an interaction was documented in the Liverpool database.

Among 11,337 COVID-19 drug–Co-medication–AE combinations, 424 (176 unique COVID-19 drug-Co-medication pairs) were significant. Among the 424 combinations, there were 53 AEs, 13 COVID-19 drugs, and 60 co-medications (Supplementary Table S1). Figure 2 depicts a network of significant DDIs, displaying the overall DDI patterns at a glance.

**FIGURE 3:**
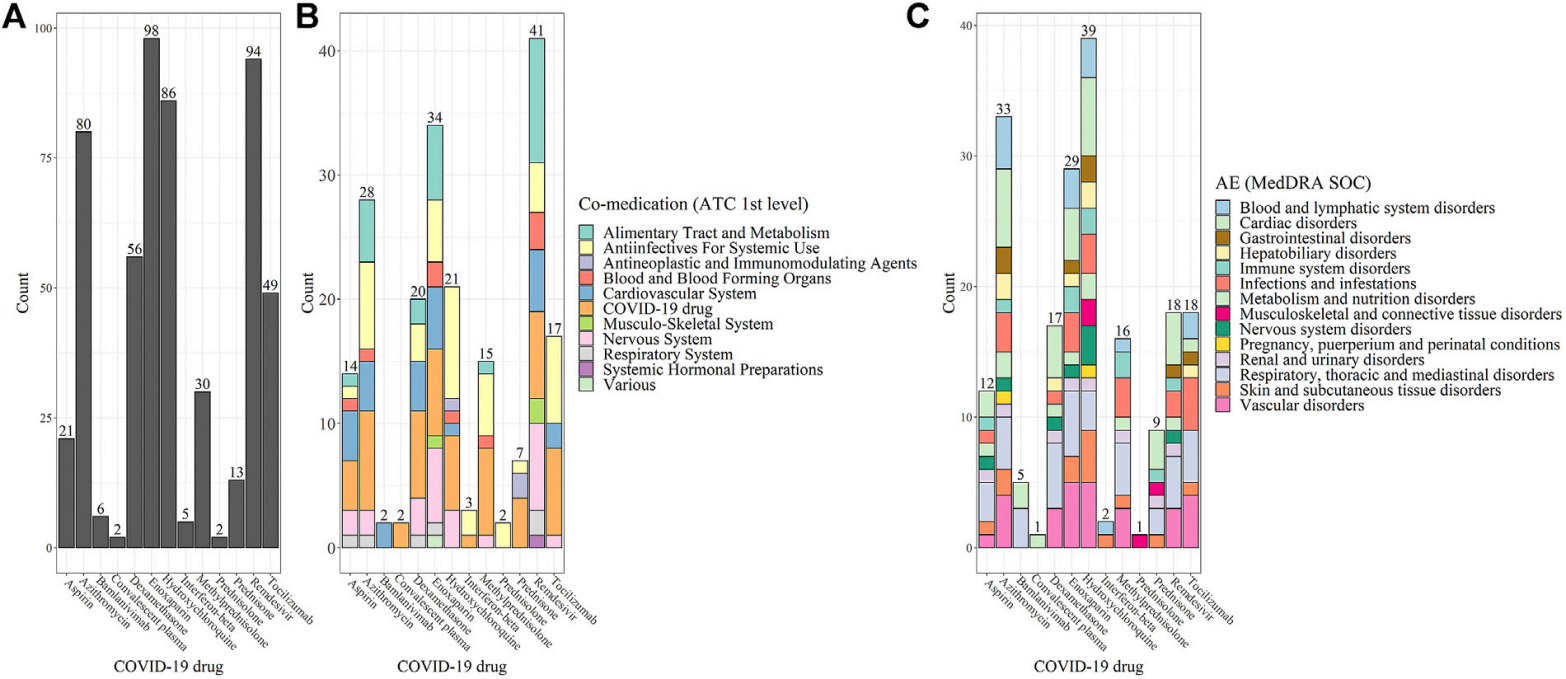
**(A)** The number of significant DDIs. **(B)** The number of unique co-medications that caused interactions with the COVID-19 drugs. **(C)** The number of unique AEs.

**FIGURE 4:**
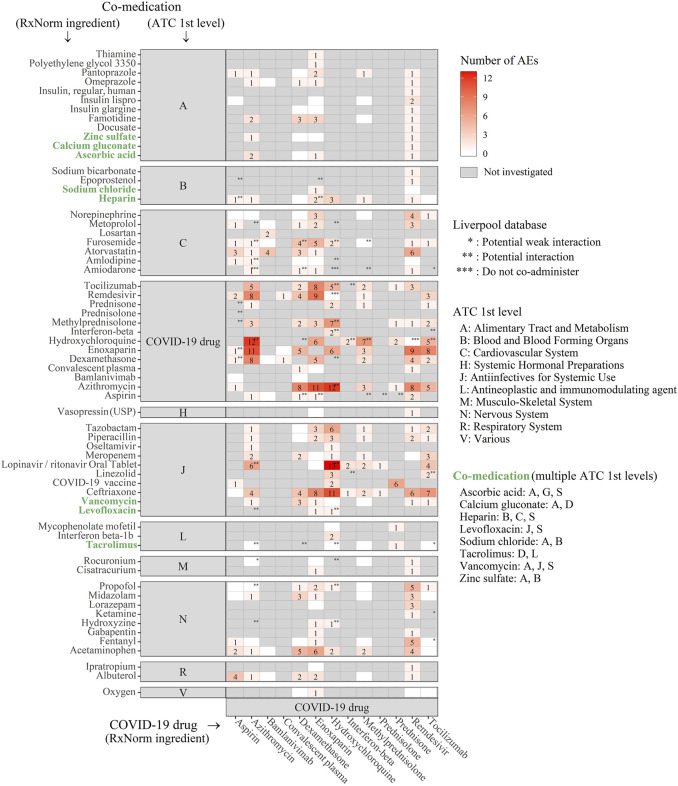
Heatmap depicting statistically significant associations between the COVID-19 drugs (on the bottom) and the co-medications (clustered by ATC 1st level, on the left). The cells were colored white to red according to the number of AEs present (grey if there was insufficient data in the FAERS database to investigate associations). Asterisks were used to denote the DDIs in the Liverpool database (*: potential weak interaction, **: potential interaction, and ***: do not co-administer).

**FIGURE 5:**
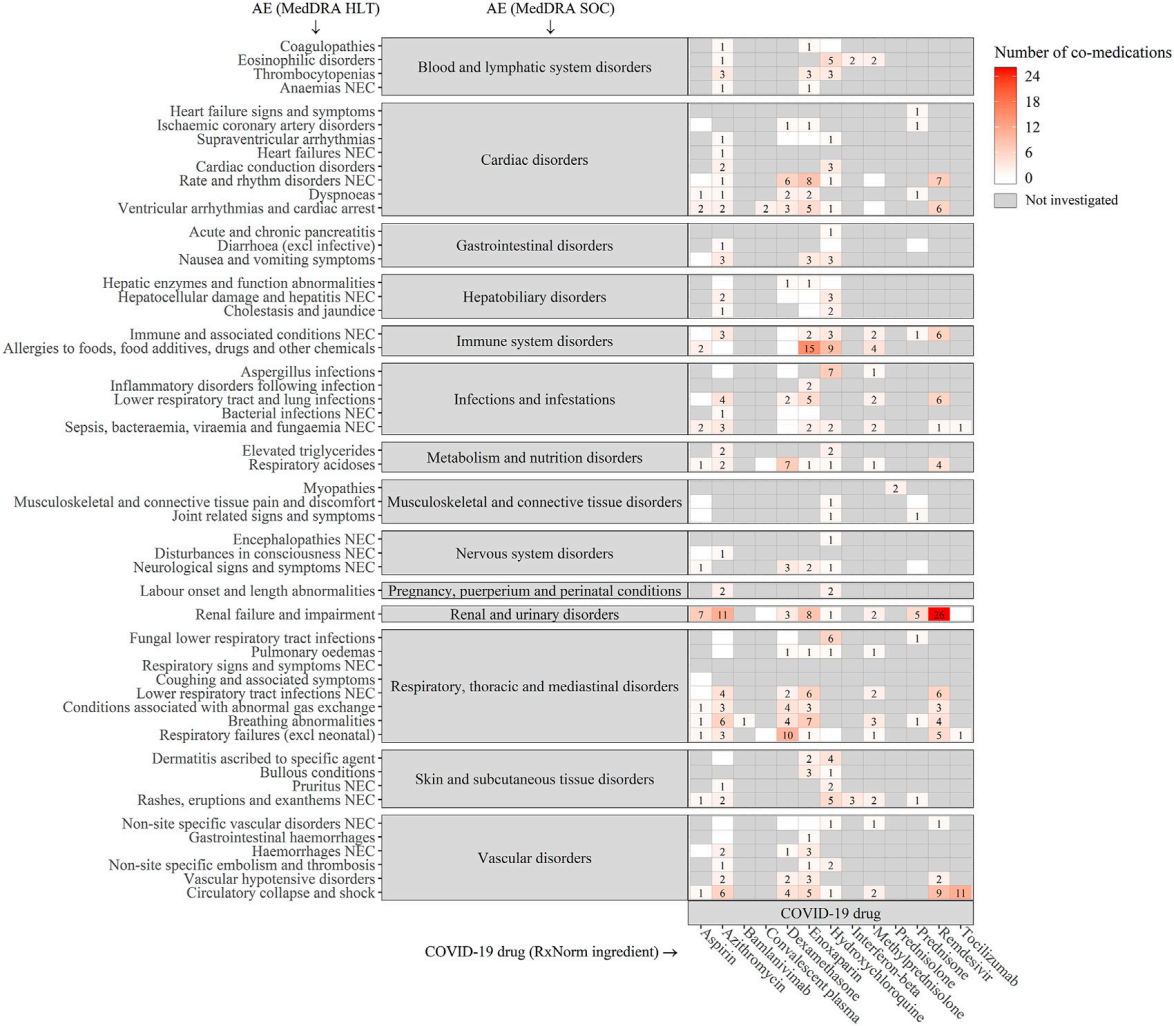
Heat map depicting statistically significant associations between the COVID-19 drugs (on the bottom) and MedDRA HLTs (clustered by MedDRA SOC, on the left). The cells were colored white to red according to the number of drugs in the co-medications that caused an AE when combined with a drug in the COVID-19 drugs (grey if there was insufficient data in the FAERS database to investigate associations).

Of 424 significant drug-drug-AEs*, enoxaparin* was ranked as the top 1 (98 combinations [26.5%]), followed by *remdesivir* (94 [22.2%]) and *hydroxychloroquine* (86 [20.3%]) (Figure 3A). *Remdesivir* interacted with 41 concomitant drugs, which is the highest number (Figure 3B and Figure 4), while *hydroxychloroquine* was affiliated with the highest number of unique AEs (39 AEs) (Figure 3C and Figure 5). The heat maps in Supplementary Figures depict all the significant drug-drug-AEs for each COVID-19 drug.

Among the 60 unique co-medications, *Alimentary Tract and Metabolism* drugs (ATC 1^st^ level: A) had the largest number of interactions with COVID-19 drugs (11 drugs [18.3%]). Among the 53 unique AEs, *renal failure and impairment* was the most prevalent AEs (56 [13.2%]).

Elderly patients exhibited a higher chance of being involved in DDIs than younger patients (176 vs 78 DDIs). Meanwhile, male patients were more vulnerable to DDIs than females (222 vs 101 DDIs) (Supplementary Table S1). The five DDIs most related to elderly and young and male and female patients are shown in Tables 2 and 3, respectively.

**TABLE 2:** The five highest and lowest adjusted odds ratios (OR) of the DDI risk associated with age.

COVID-19 drug	Co-medication	MedDRA HLT	<65	≥65	OR^a^ (CI) (≥65)
Highest OR					
Azithromycin	Hydroxychloroquine	Heart failures NEC	13	18	1.98 (1.44–2.73)
Hydroxychloroquine	Lopinavir/ritonavir Oral Tablet	Encephalopathies NEC	5	23	1.79 (1.24–2.59)
Hydroxychloroquine	Piperacillin	Dermatitis ascribed to specific agent	12	14	1.73 (1.20–2.48)
Tocilizumab	Meropenem	Aspergillus infections	24	12	1.72 (1.3–2.29)
Enoxaparin	Hydroxychloroquine	Dermatitis ascribed to specific agent	15	16	1.72 (1.2–2.47)
Lowest OR					
Azithromycin	Ceftriaxone	Labor onset and length abnormalities	35	0	0.01 (0–0.09)
Hydroxychloroquine	Oseltamivir	Labor onset and length abnormalities	26	0	0.01 (0–0.1)
Hydroxychloroquine	Ceftriaxone	Labor onset and length abnormalities	33	0	0.01 (0–0.1)
Azithromycin	Oseltamivir	Labor onset and length abnormalities	25	0	0.07 (0–0.11)
Hydroxychloroquine	Ceftriaxone	Elevated triglycerides	27	6	0.31 (0.18–0.55)

a Adjusted for gender, number of drug exposures, use of COVID-19, drug, and use of co-medication.

**TABLE 3:** The five highest and lowest adjusted odds ratios (OR) of the DDI risk associated with gender.

COVID-19 drug	Co-medication	MedDRA HLT	Female	Male	OR^a^ (CI) (male)
Highest OR					
Aspirin	Atorvastatin	Sepsis, bacteraemia, viraemia, and fungaemia NEC	3	26	1.09 (1.08–1.1)
Azithromycin	Furosemide	Sepsis, bacteraemia, viraemia, and fungaemia NEC	5	24	1.09 (1.07–1.1)
Enoxaparin	Azithromycin	Sepsis, bacteraemia, viraemia, and fungaemia NEC	14	41	1.08 (1.07–1.09)
Aspirin	Heparin	Sepsis, bacteraemia, viraemia, and fungaemia NEC	2	25	1.08 (1.07–1.09)
Hydroxychloroquine	Ceftriaxone	Bullous conditions	16	10	1.08 (1.07–1.09)
Lowest OR					
Tocilizumab	Ceftriaxone	Eosinophilic disorders	7	22	0.87 (0.82–0.92)
Hydroxychloroquine	Ceftriaxone	Eosinophilic disorders	27	35	0.89 (0.84–0.95)
Hydroxychloroquine	Lopinavir/ritonavir Oral Tablet	Eosinophilic disorders	34	45	0.91 (0.86–0.97)
Methylprednisolone	Hydroxychloroquine	Eosinophilic disorders	13	12	0.91 (0.86–0.96)
Interferon-beta	Lopinavir/ritonavir Oral Tablet	Eosinophilic disorders	14	25	0.92 (0.86–0.98)

a Adjusted for age, number of drug exposures, use of COVID-19, drug, and use of co-medication.

### 3.3 DDIs assessment

The Liverpool database covers interactions between 34 COVID-19 drugs and 572 co-medications, with 282 potential weak interactions, 1,382 potential interactions, and 248 do not co-administer as of 26 January 2022. Since the Liverpool database lacks information on specific adverse events, we compared our findings to the Liverpool database using drug-drug level data (176 drug-drug pairs) rather than drug-drug-AE level data (424 drug-drug-AE combinations).

Of the 176 significant drug-drug pairs, 20 DDIs (19 potential interactions and 1 do not co-administer) were documented in the Liverpool database, 135 DDIs were determined to be non-significant by the Liverpool database, and the remaining 21 DDIs lacked relevant information in the Liverpool database (Supplementary Table S1 and Figure 4).

The empirical *p*-value generated by Monte Carlo simulations was approximately 0.001.

## 4 Discussion

This retrospective study examined potential DDIs with drugs used for COVID-19 treatment using the spontaneous reporting system database and compared the findings to the DDI source in the Liverpool database in order to identify previously unrecognized interactions. According to the results of the Monte Carlo simulation, the detected drug-drug interactions are most likely not coincidental (*p*-value = 0.001). Thus, there is a high likelihood of developing clinical trial hypotheses based on the detected novel DDIs.

Some of our findings are consistent with DDIs documented in the Liverpool database and other published studies. According to Nguyen et al. (Nguyen et al., 2020), *hydroxychloroquine* and *azithromycin*, alone or in combination, were suspected to be associated with prolonged QT and/or ventricular tachycardia. We detected that *hydroxychloroquine* and *azithromycin* interaction was related to *cardiac conduction disorders.* In addition, *hydroxychloroquine-levofloxacin* and *hydroxychloroquine-lopinavir/ritonavir* interactions detected by our methods were confirmed by the published finding - co-prescribing *levofloxacin or lopinavir/ritonavir* with *hydroxychloroquine* can result in a dramatic increased risk of cardiotoxicity (Cattaneo et al., 2020; Singh et al., 2021). Meanwhile, QT prolongation is associated with the interaction between *azithromycin* and *lopinavir/ritonavir* (Samarendra et al., 2001; Zequn et al., 2021). As demonstrated in our findings, the interaction between *azithromycin* and *lopinavir/ritonavir* may be associated with *Cardiac conduction disorders.*

Notably, we included well-known interactions as well as significant DDIs that were previously deemed to have no interaction in the Liverpool database. For example, while the Liverpool database indicated that there was no interaction between *azithromycin* and *ceftriaxone,* there is a case report about a heart transplant recipient who developed elevated tacrolimus blood levels following the administration of intravenous *azithromycin* and *ceftriaxone* (Shullo et al., 2010). Additionally, we discovered an interaction between *dexamethasone* and *vancomycin,* and treatment failures for adult pneumococcal meningitis were reported in adults receiving standard *vancomycin* doses with adjunctive *dexamethasone* (Viladrich et al., 1991; de Gans and Van de Beek, 2002). Also, our findings indicated that the interaction between *dexamethasone* and *albuterol* resulted in *Rate and rhythm disorders NEC*, despite the fact that the Liverpool database determined that this interaction had no adverse effects. The concomitant use of beta-2 adrenergic agonists and corticosteroids may have additive hypokalemic effects (Taylon et al., 1992). Since beta-2 agonists can sometimes cause QT interval prolongation (Wong et al., 1990), the development of hypokalemia may increase the risk of ventricular arrhythmias, including torsade de pointes. Combining *hydroxychloroquine* and *linezolid* may increase the risk or severity of nerve damage, which is a potential side effect of both medications (Argov and Mastaglia, 1979; Carrion et al., 1995), and this interaction was significant in our findings. The interaction between *hydroxychloroquine* and *heparin* resulted in *Non-site specific embolism and thrombosis* and *Thrombocytopenias*, and *hydroxychloroquine* which interferes with platelet aggregation reactions (Cornwell et al., 2021) (the main hemostatic defense of heparinized patients (Mohammad et al., 1981)) may induce bleeding when it is used in patients receiving *heparin*.

Additionally, our findings included novel DDIs not previously investigated in the Liverpool database. For instance, the Liverpool database did not contain information about the interaction between *azithromycin* and *ascorbic acid*, but our findings indicated that this interaction was associated with *Respiratory acidoses*. Although no pharmacokinetic interaction between these two drugs is known at the moment, there are 3 case reports of COVID-19 patients developing adverse drug reactions such as supraventricular tachycardia, chest discomfort, headache, sinus tachycardia, dyspnoea, and abdominal pain after receiving combinations of COVID-19 therapies including *azithromycin* and *ascorbic acid* (Darko, 2021).

We rely upon the Liverpool database to evaluate our findings because it is currently one of the most reliable databases containing information on COVID-19 DDIs. However, a recent study found that if clinicians relied solely on the Liverpool database, a substantial number of potentially significant DDIs may be overlooked (Biswas and Roy, 2021). It should be noted that many of the DDIs listed in the Liverpool database are theoretical and have not been demonstrated to cause harm to patients. In this project, we detected DDI signals which were confirmed by literature, however, those signals were previously deemed to have no interaction in the Liverpool database. Our study investigated a broader range of drug combinations than the Liverpool database did (34 COVID-19 drugs-1,420 co-medications vs 34 COVID-19 drugs-572 co-medications), allowing us to identify many novel DDIs that were not previously depicted in the Liverpool database.

We further identified age- and sex-related differences in drug-drug interaction. For instance, the interaction between *hydroxychloroquine* and *azithromycin* was considered significant in our study and was also documented in the Liverpool database. However, we discovered that this combination was associated with a higher risk of adverse events in elder patients than in young patients, information that the Liverpool database didn’t provide. Additionally, *hydroxychloroquine* and *lopinavir/ritonavir* had an interaction in both our findings and the Liverpool database, but our finding additionally provided that this interaction was associated with a higher risk of adverse events in female patients than in male patients.

Our project is a pilot study, and there are limitations that we want to acknowledge as guidelines for further studies on the DDI research using the FAERS database. First, although post-marketing pharmacovigilance databases serve an important role, there is no certainty that the reported adverse event was due to the drug since the FAERS database does not include patient characteristics, their medical history, dosage information, or route information (eye drop or pill), which are important risk factors of the occurrence of drug-drug interactions (Romagnoli et al., 2017). The FDA does not demand proof of a causal relationship between a drug and an incident, and reports may not always provide sufficient information to evaluate an event. For example, even though we found the interaction between *aspirin* and *enoxaparin* may be related to *Renal failure and impairment,* (Shullo et al., 2002), renal impairment might be caused by a patient’s medical condition, such as COVID-19 or sepsis, rather than by DDI. Additionally, this could have represented a drug-disease interaction since enoxaparin is removed by the kidneys, and renal failure or impairment would increase enoxaparin levels and the risk of AEs, such as bleeding. Due to this limitation, we further note that while we controlled for potentially confounding demographic factors and the number of drug exposures, we did not account for other potentially significant confounding variables such as smoking behaviors and general health state. Second, reporting bias, such as under-reporting or selective reporting of ADRs, limits the utility of using the FAERS database to detect actual DDI signals (Ghosh and Dewanji, 2015; Noguchi et al., 2021). An analysis of 37 studies showed that 94 percent of ADR occurrences were not recorded (Hazell and Shakir, 2006). Unknown causality, the ADR being insignificant, or the ADR being too well known are potential causes for the reporting bias. Third, the quality of the FAERS database needs further improvement. For instance, there are many duplicative reports and some reports are incomplete in the FAERS data. The data cleaning and missing value imputation can be potential solutions.

## 5 Conclusion

This work contributes to the current range of evidence regarding DDIs in patients with COVID-19 by examining DDIs with a high number of adverse reports. Our exploratory data analysis for hypothesis generation presented insight into the DDIs associated with COVID-19 drugs for subsequent confirmatory analysis, which involves additional validation studies for the drugs’ pharmacokinetic properties, metabolic pathways, and pharmacodynamics.

## Data Availability

Publicly available datasets were analyzed in this study. This data can be found here: https://fis.fda.gov/extensions/FPD-QDE-FAERS/FPD-QDE-FAERS.html
