# Supplementary material for: Detecting drug-drug interactions between therapies for COVID-19 and concomitant medications through the FDA adverse event reporting system
Source: Front Pharmacol. 2022 Jul 22;13:938552. doi: 10.3389/fphar.2022.938552 (PMC9353301; doi:10.3389/fphar.2022.938552)
Supplement: Supplementary file 1 [file DataSheet2.docx]

**Detecting Drug-Drug interactions between Therapies for COVID-19 and Concomitant Medications through the FDA Adverse Event Reporting System**

**Eugene Jeong, MS^1^, Scott D Nelson, PharmD, MS^1^, Yu Su, PhD^5^, Bradley Malin, PhD^1,3,4^, Lang Li, PhD^2^, You Chen, PhD^1,4^**

^1^Department of Biomedical Informatics, School of Medicine, Vanderbilt University Medical Center, Nashville, Tennessee, United States

^2^Department of Biomedical Informatics, College of Medicine, The Ohio State University, Columbus, Ohio, United States

^3^Department of Biostatistics, School of Medicine, Vanderbilt University Medical Center, Nashville, Tennessee, United States

^4^Department of Computer Science, School of Engineering, Vanderbilt University, Nashville, Tennessee, United States

^5^Department of Computer Science and Engineering, College of Engineering, The Ohio State University, Columbus, Ohio, United States

*** Correspondence:**

You Chen, Ph.D.

email: you.chen@vanderbilt.edu

Keywords: drug-drug interactions_1_, COVID-19_2_, FAERS_3_, hypothesis generation_4_, logistic regression_5,_ additive interaction_6_, multiplicative interaction_7_, discovery-driven_8_ .


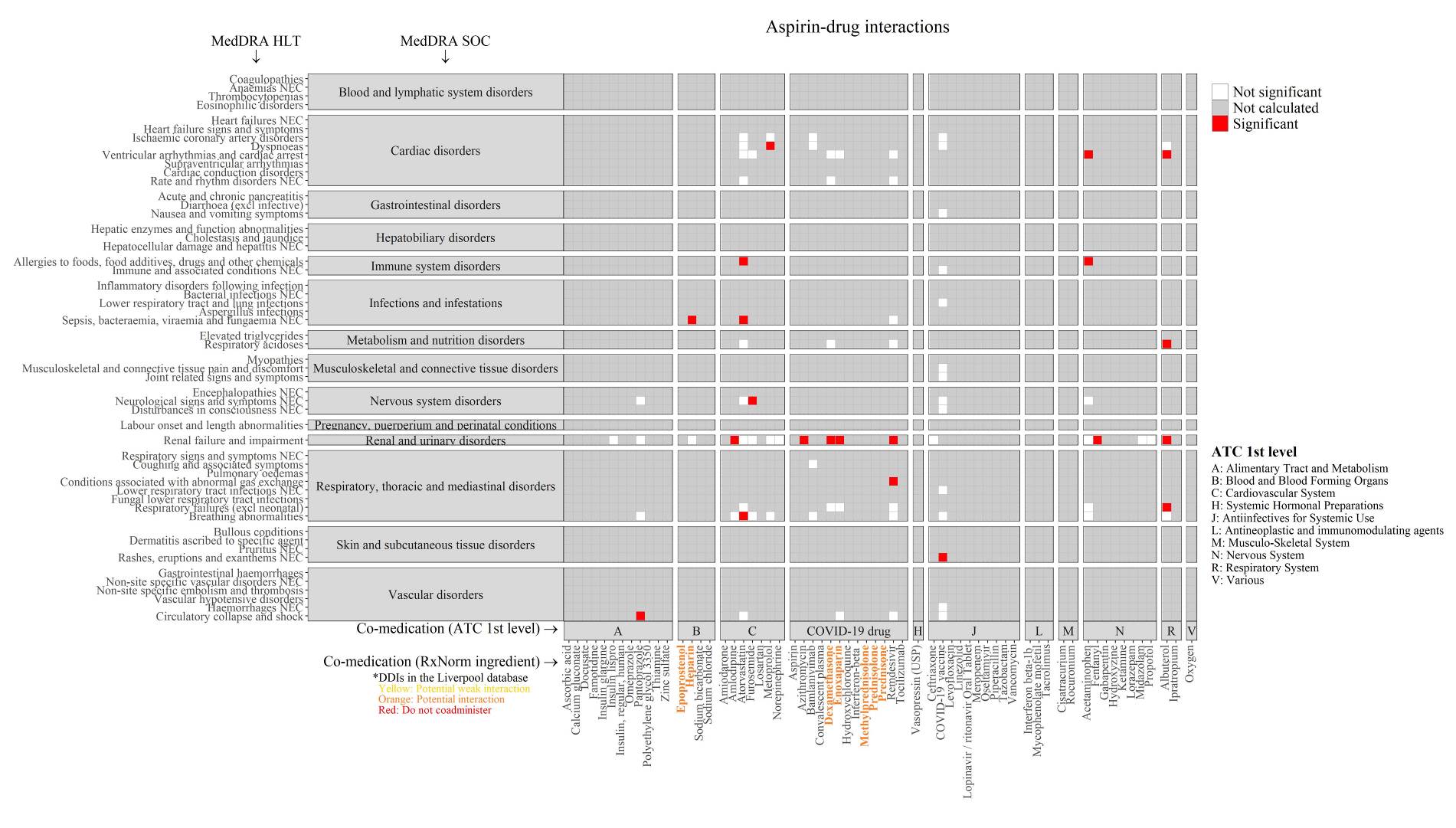


Supplementary Figure 1. Potential aspirin-co-medication interactions


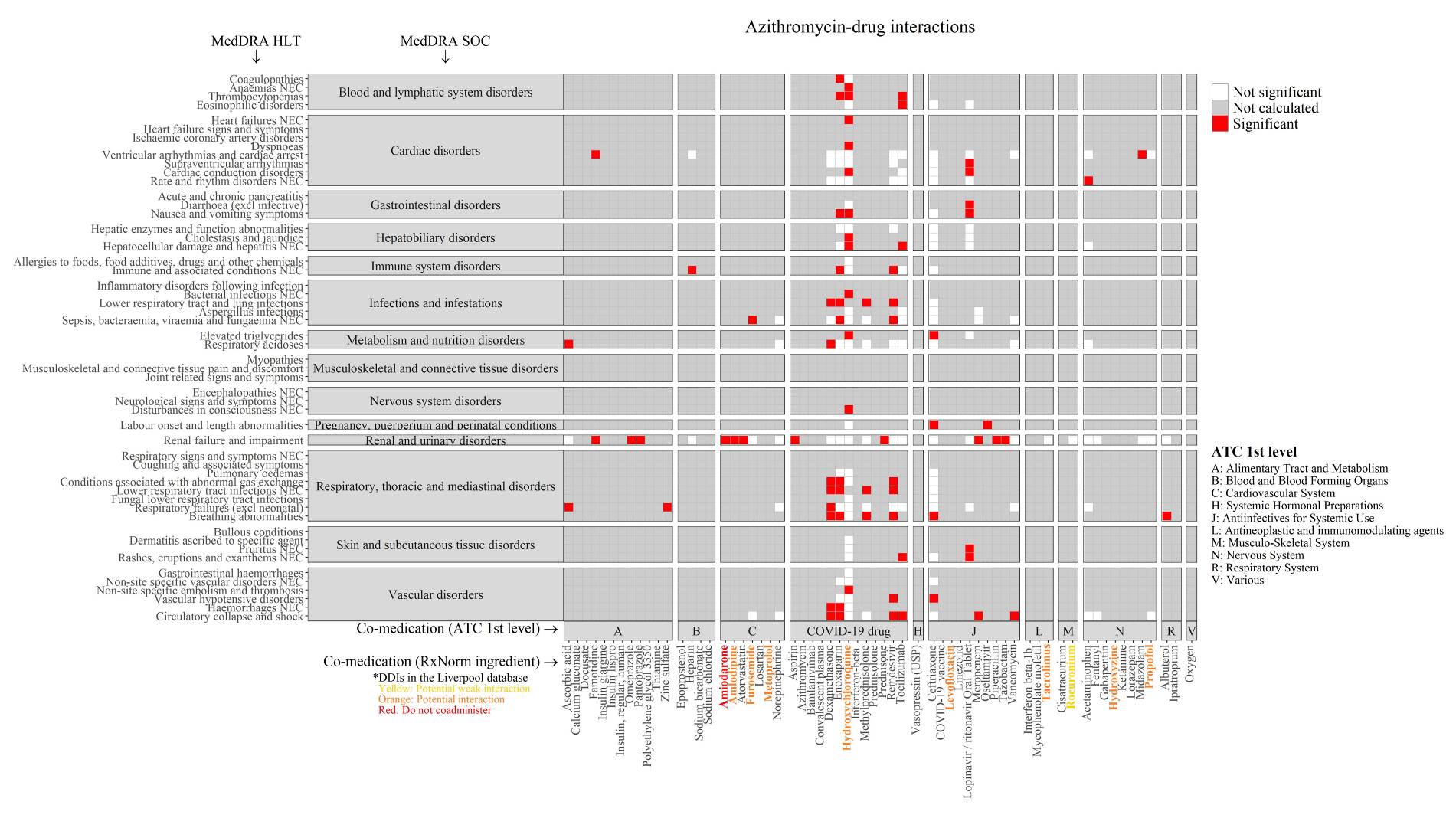


Supplementary Figure 2. Potential azithromycin-co-medication interactions


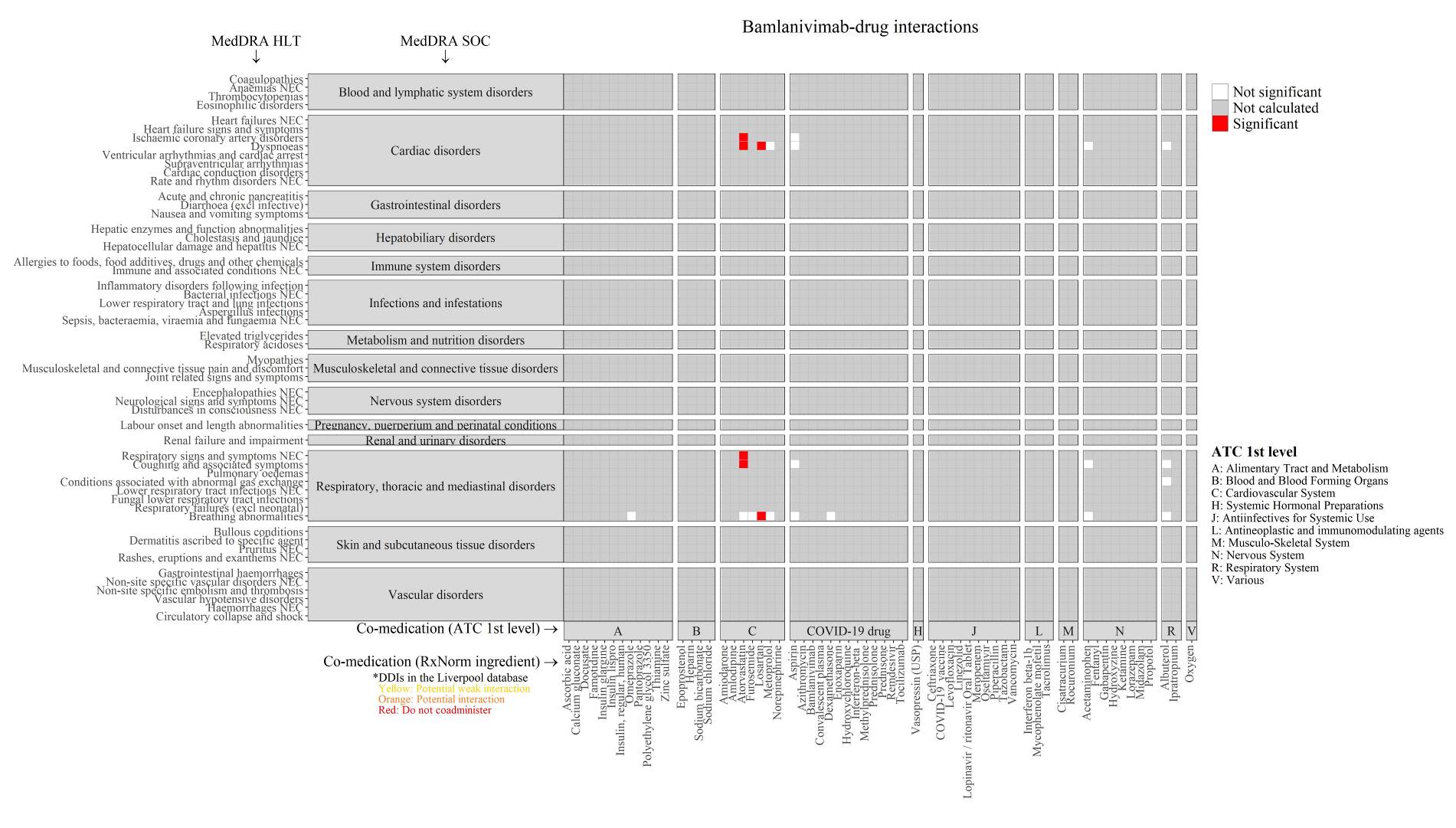


Supplementary Figure 3. Potential bamlanivimab-co-medication interactions


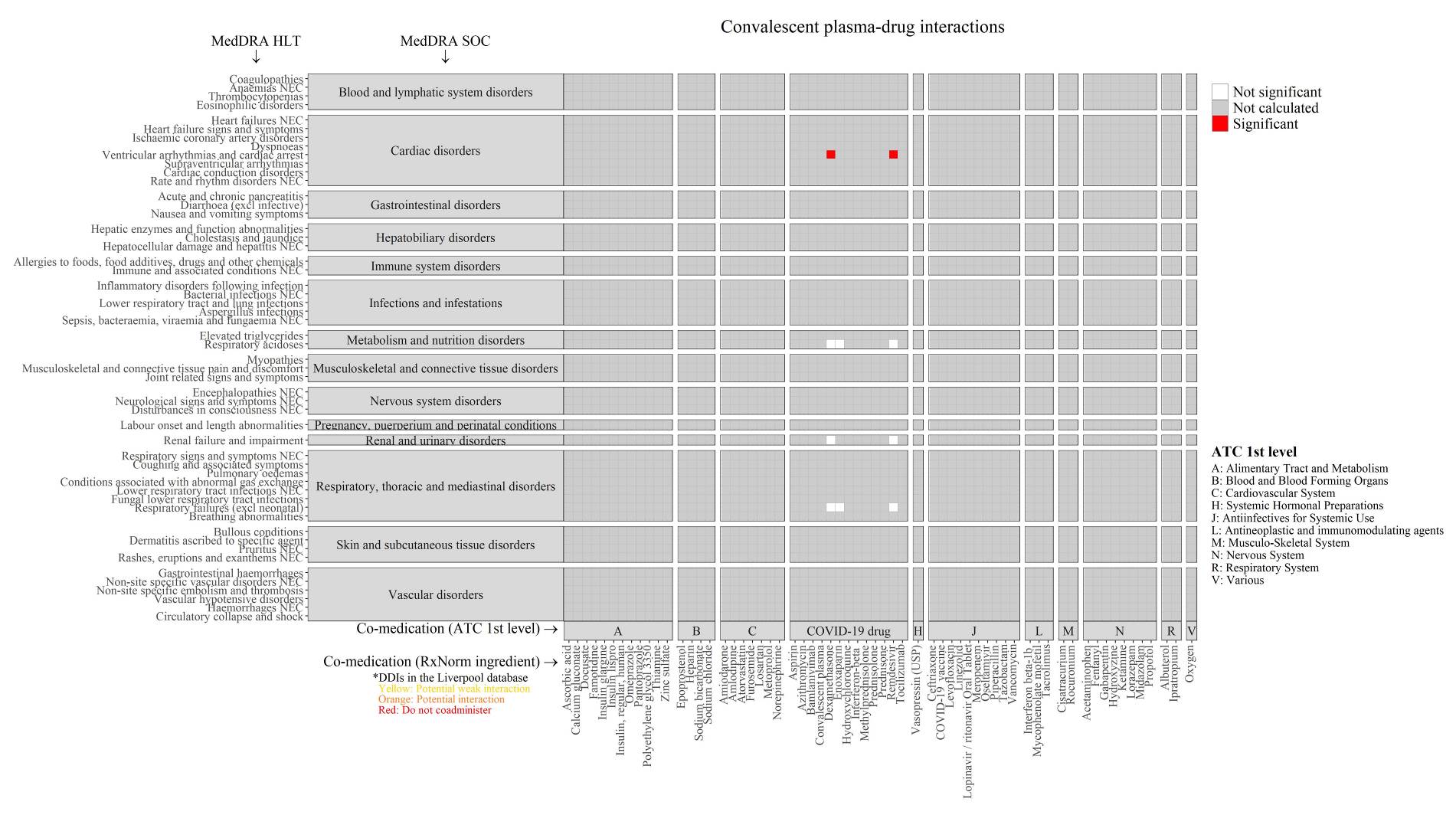


Supplementary Figure 4. Potential convalescent plasma-co-medication interactions


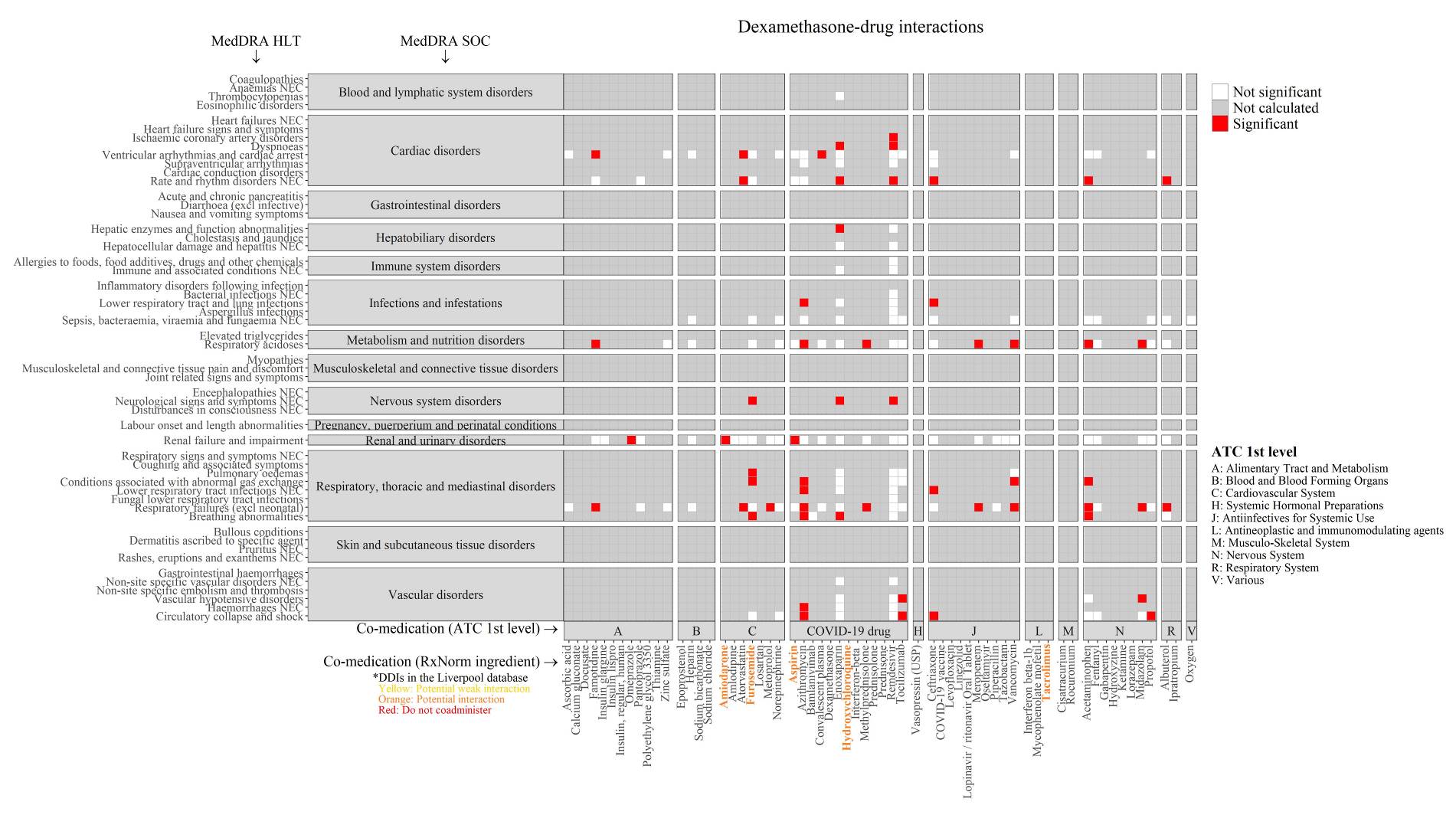


Supplementary Figure 5. Potential dexamethasone-co-medication interactions


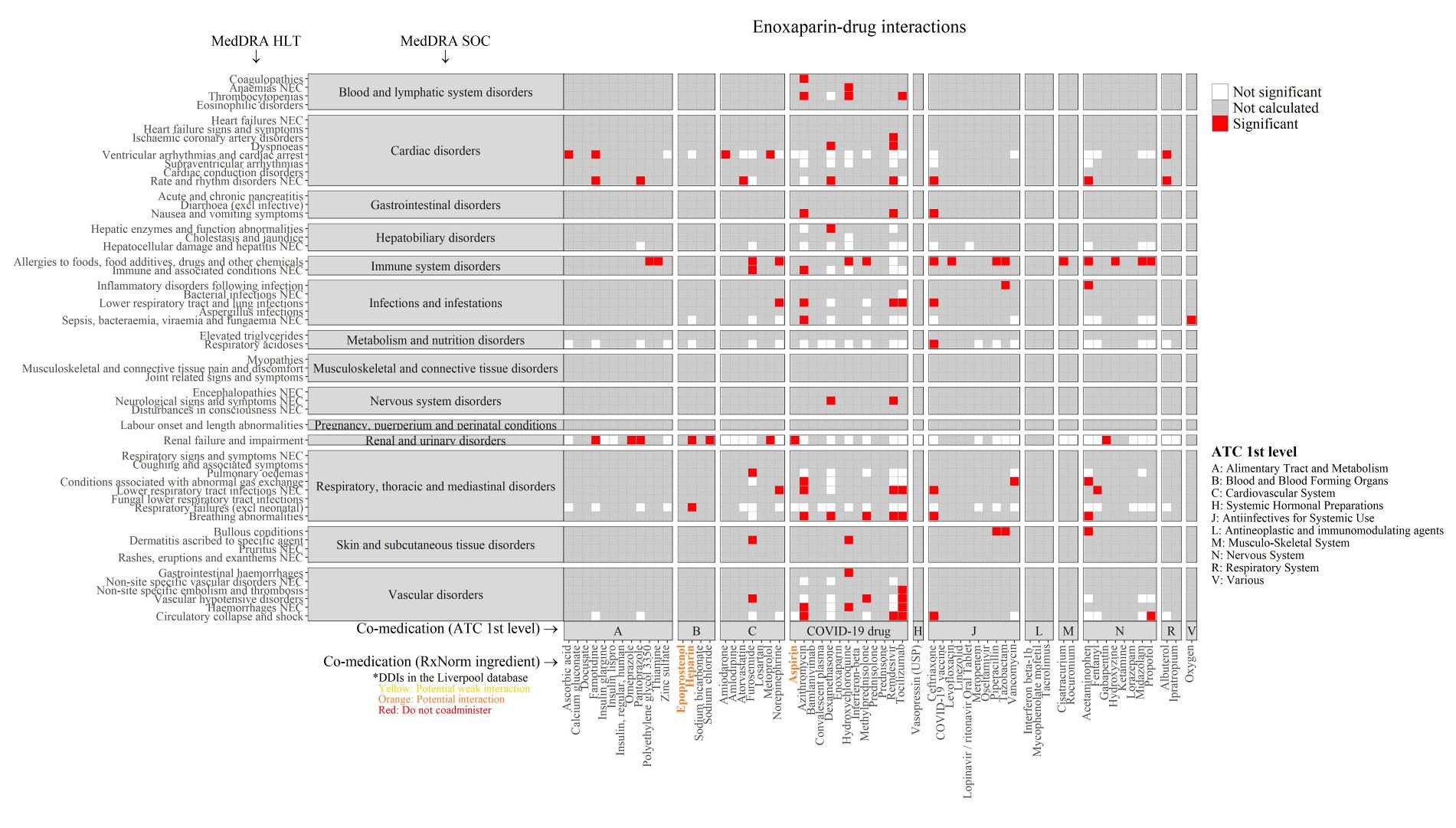


Supplementary Figure 6. Potential enoxaparin-co-medication interactions


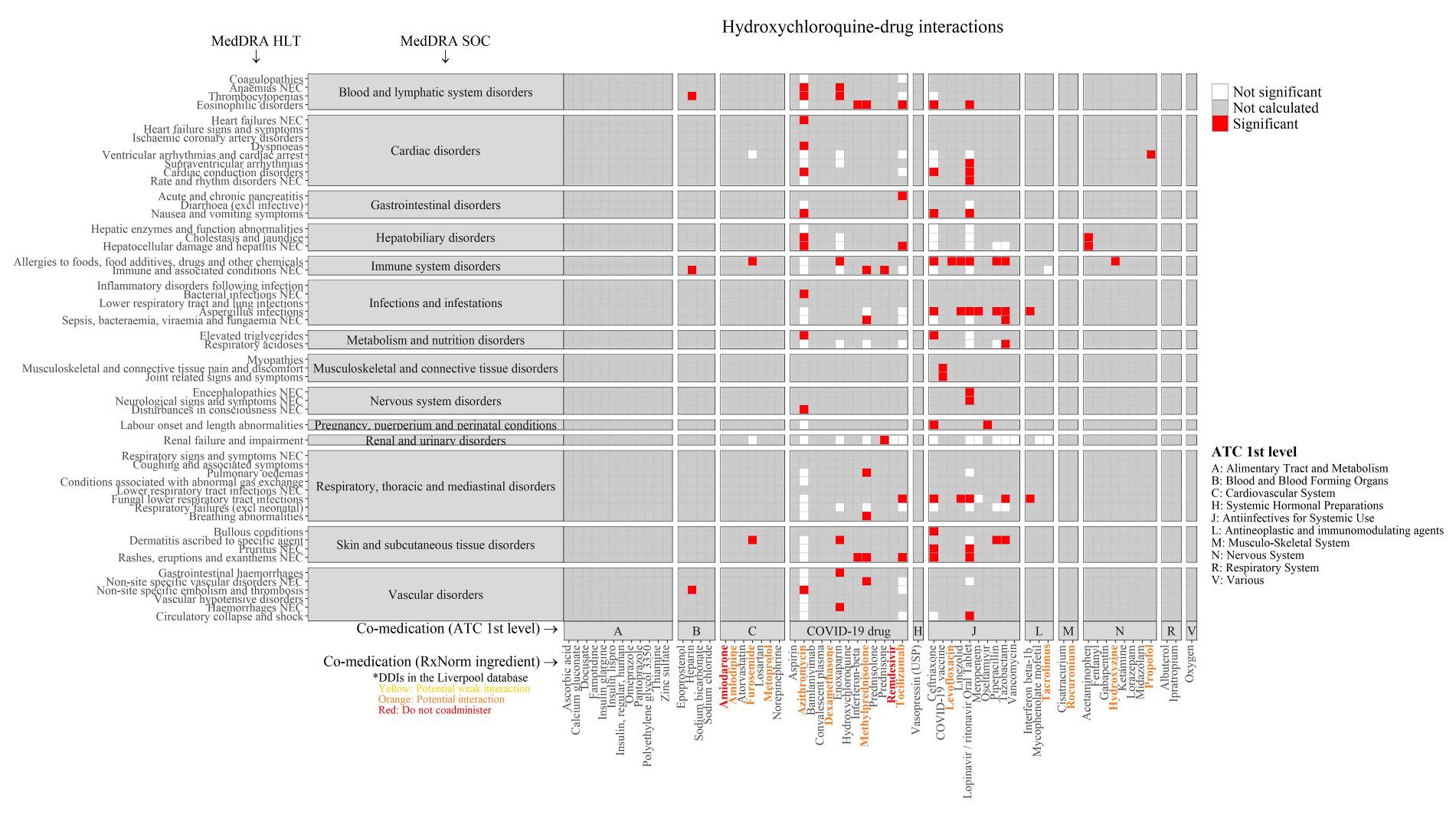


Supplementary Figure 7. Potential hydroxychloroquine-co-medication interactions


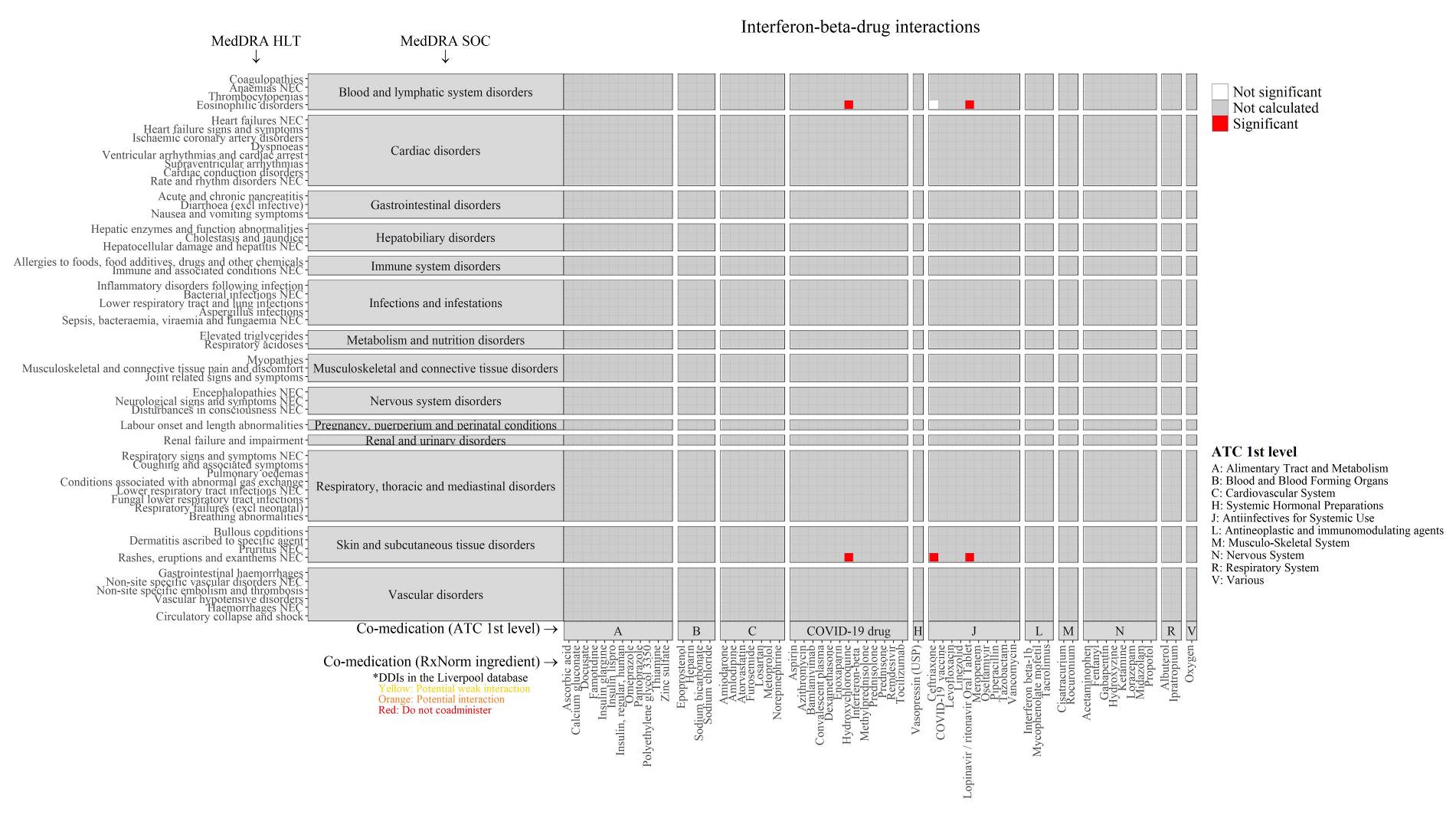


Supplementary Figure 8. Potential interferon-beta-co-medication interactions


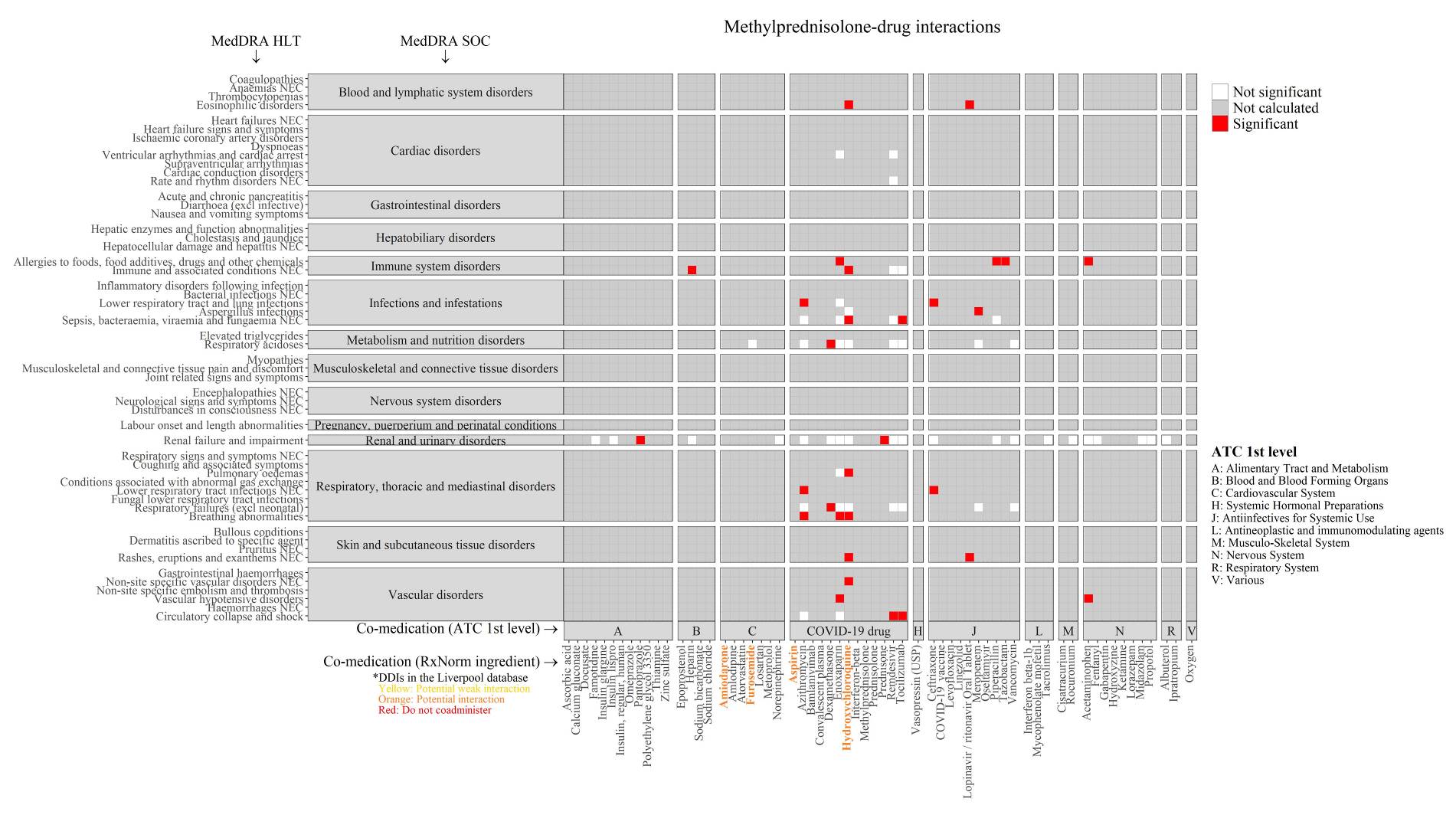


Supplementary Figure 9. Potential methylprednisolone-co-medication interactions


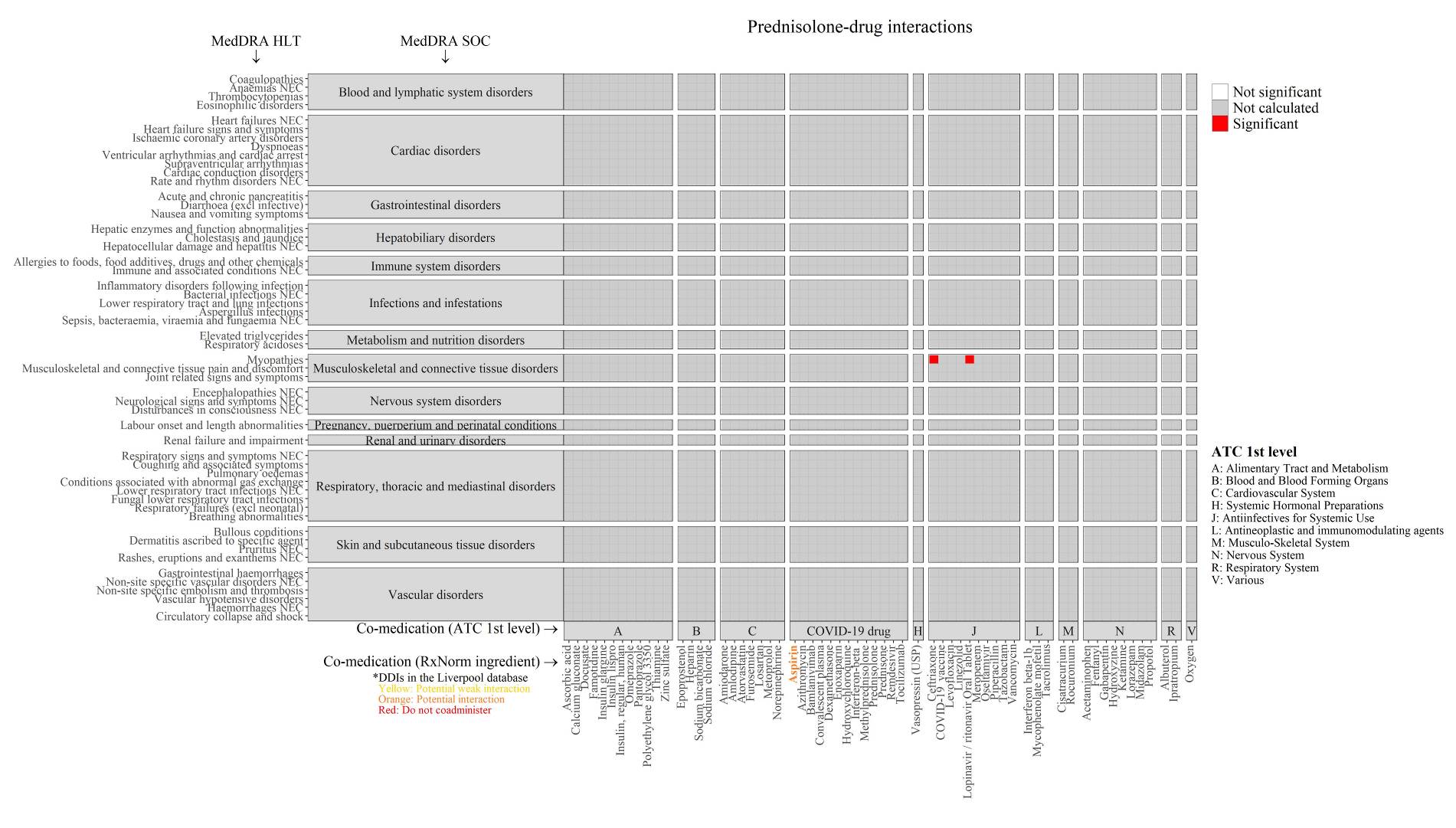


Supplementary Figure 10. Potential prednisolone-co-medication interactions


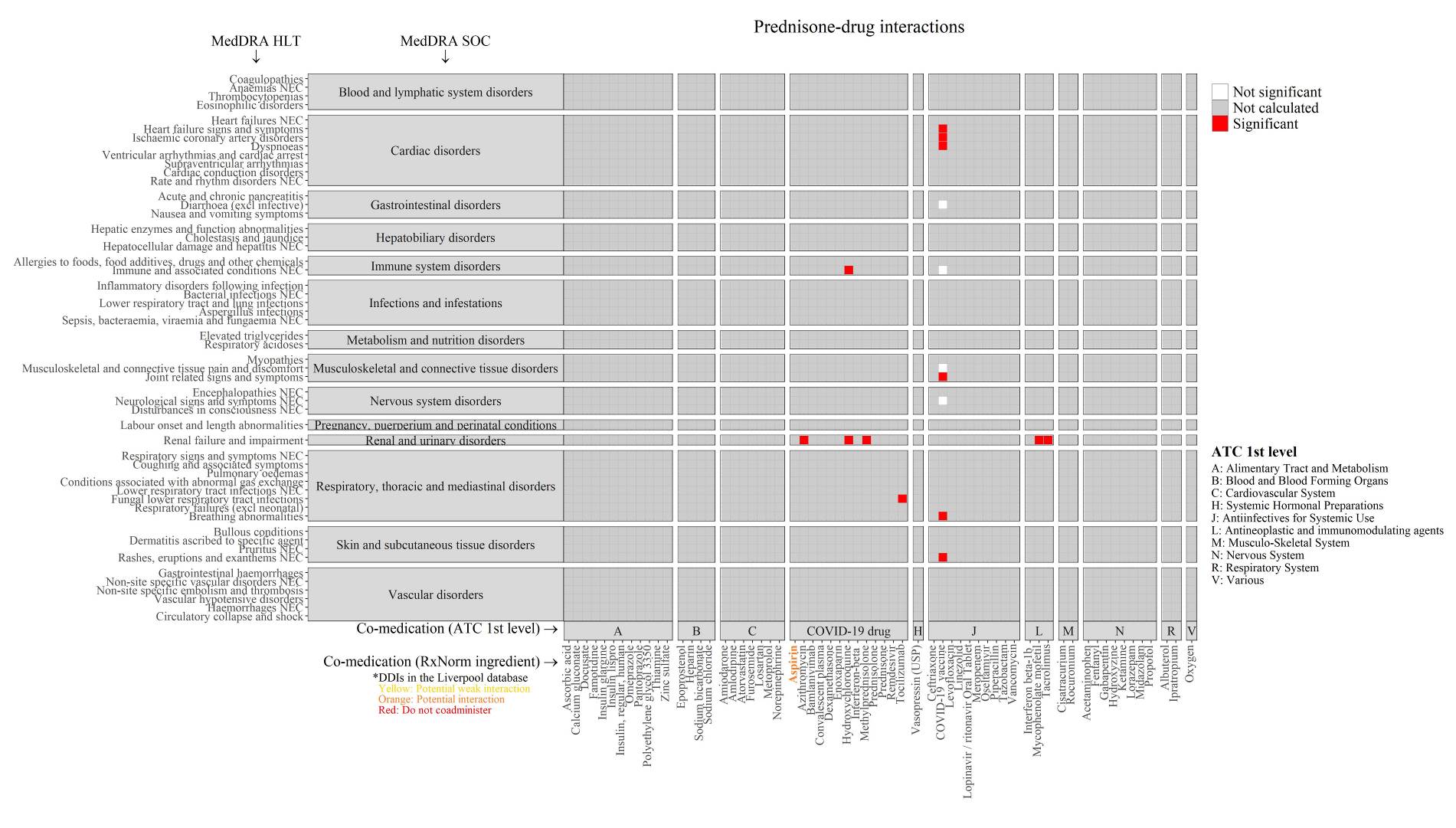


Supplementary Figure 11. Potential prednisone-co-medication interactions


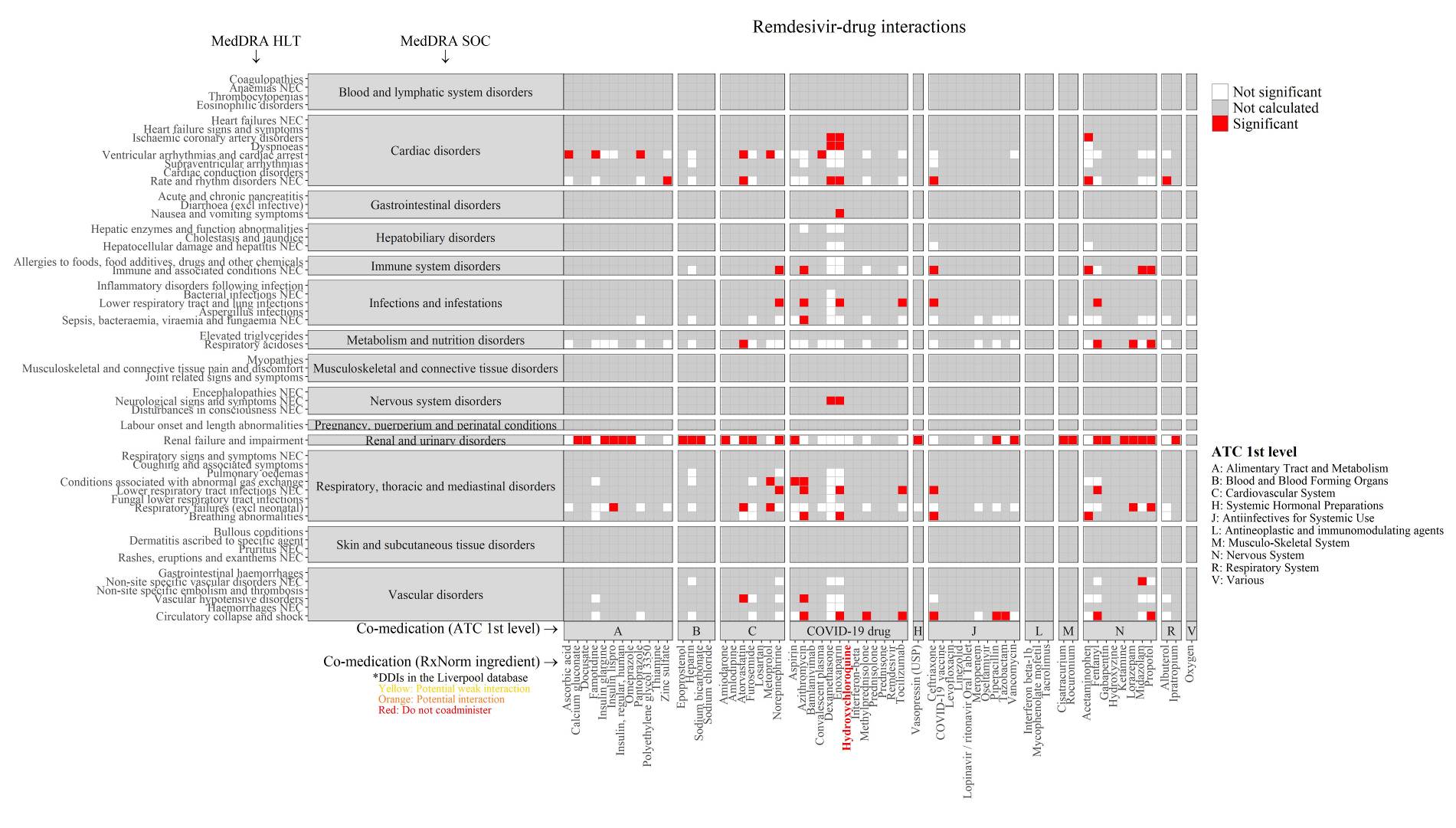


Supplementary Figure 12. Potential remdesivir-co-medication interactions


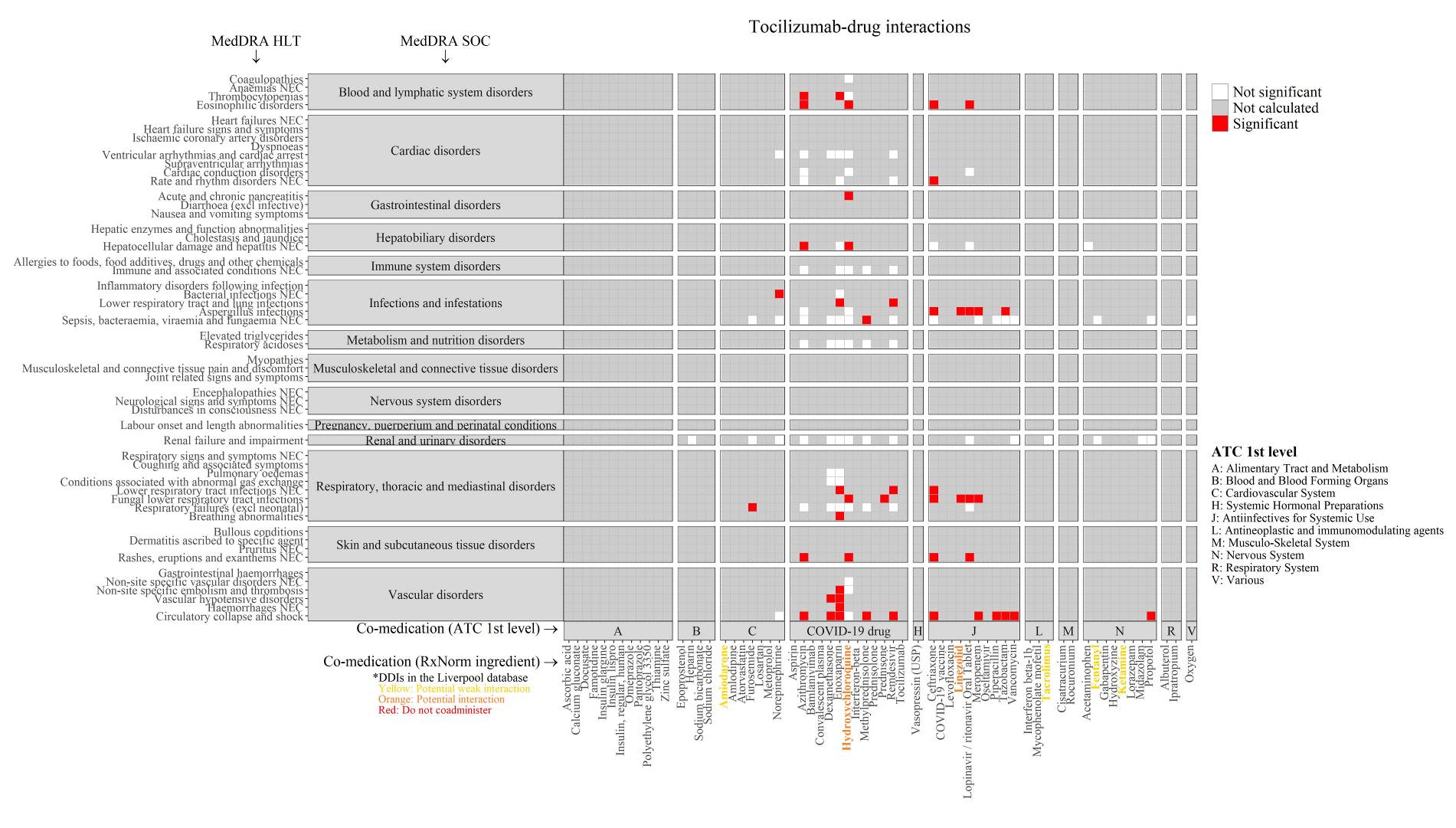


Supplementary Figure 13. Potential tocilizumab-co-medication interactions
